# Closing the gap between trial and clinical reality in long-term left ventricular assist device use outcomes

**DOI:** 10.1093/eschf/xvag181

**Published:** 2026-06-24

**Authors:** Federica Guidetti, Mateusz Sokolski

**Affiliations:** Department of Clinical Science and Education, Södersjukhuset, Karolinska Institute, 171 77 Stockholm, Sweden; Department of Cardiology, University Cardiovascular Center, Bern University Hospital, Inselspital, 3010 Bern, Switzerland; Clinic of Cardiac Transplantation and Mechanical Circulatory Support, Faculty of Medicine, Wroclaw Medical University, Institute of Heart Diseases, 50-556 Wroclaw, Poland; Institute of Heart Diseases, University Hospital, 50-556 Wroclaw, Poland

**Keywords:** Advanced heart failure, Left ventricular assist device, HeartMate 3, Heart transplantation, Mechanical circulatory support, Long-term outcomes

**This article refers to Long-term outcomes of patients implanted with HeartMate3 left ventricular assist device—a real-world, single-centre, observational study by S. Legtenberg *et al.* ESC Heart Fail. 2026 Mar 3;13(2):xvag074. https://doi.org/10.1093/eschf/xvag074.**

Heart failure (HF) remains a major global health challenge. Despite substantial advances in life-saving, evidence-based medical and device therapies, ∼10%–15% of patients progress to advanced HF (AdvHF).^1^ This stage is characterized by severe symptoms, recurrent hospitalizations, poor functional capacity, intolerance to guideline-directed medical therapy, and an estimated 1-year mortality exceeding 40%.^2,3^ Consequently, current expert statements strongly recommend timely referral of these patients to specialized centres for advanced therapies evaluation.^4^ Although heart transplantation (HTx) remains the gold standard treatment for eligible AdvHF patients, providing a median survival of 10–13 years, persistent donor shortage and contraindications to HTx, have increased the use of left ventricular assist device (LVAD).^5^

The introduction of the fully magnetically levitated centrifugal-flow HeartMate 3™ (HM3) device represented a major milestone in the field. By improving haemocompatibility, HM3 technology has substantially decreased the risks of pump thrombosis, stroke, and device malfunction. In the MOMENTUM 3 trial, HM3 demonstrated superiority over the previous-generation axial-flow device in terms of survival free from disabling stroke or reoperation for device dysfunction.^6^ Long-term follow-up from the trial reported HM3 survival rates of 87%, 79%, and 58% at 1, 2, and 5 years, respectively. Nonetheless, despite these advances, long-term real-world data, particularly from European cohorts, remain limited.

In this context, the study by Legtenberg *et al*.^7^ provides an important contribution to the growing body of real-world evidence supporting the efficacy and durability of HM3 therapy outside the highly selected North American clinical trial populations. The authors report outcomes from 176 consecutive patients implanted with HM3 at the University Medical Center Groningen (UMCG) in the Netherlands. The cohort reflects a contemporary European LVAD population, with a mean age of 56 ± 11 years, predominantly non-ischaemic HF aetiology, and advanced clinical profiles, as ∼75% of patients were classified as INTERMACS class 2 or 3. Notably, most patients underwent implantation either as bridge-to decision (BTD; 42%) or bridge-to-transplant (BTT; 24%), whereas only one-third received the device as destination therapy (DT), underscoring the differences between European and North American LVAD implantation strategies.

The reported survival outcomes are encouraging. The authors observed survival rates of 87% at 1 year, 82% at 2 years, and 61% at 5 years, closely mirroring those observed in both the MOMENTUM 3 trial and the ELEVATE registry.^8,9^ Importantly, these findings confirm that the favourable outcomes achieved in highly selected trial populations are reproducible in routine European clinical practice. At the same time, they emphasize the progressive maturation of LVAD as a durable long-term treatment strategy, shifting the clinical focus from mere survival towards quality and sustainability of survival.

One of the striking findings reported by Legtenberg *et al*. is the complete absence of pump thrombosis during follow-up. Although derived from a relatively small single-centre cohort, this observation reinforces one of the most important advances associated with HM3 therapy. Whereas previous-generation devices were significantly limited by pump thrombosis, HM3 has demonstrated a remarkably low pump thrombosis rate both in the MOMENTUM-3 trial (2.1% at 5 years) and in the ELEVATE registry (1.1% at 5 years).^9^ The low incidence of cerebrovascular events (2% within 2 years) further supports the growing evidence that HM3 has significantly improved neurological outcomes compared with earlier-generation LVADs.^6,9^

However, while the study confirms the marked reduction in device-specific and cerebrovascular complications, it also highlights the persistent burden of adverse events during long-term support. The majority of complications occurred within the first year after implantation. Device-related infections occurred at a rate of 0.29 events per patient-year, while HF hospitalizations occurred at 0.18 events per patient-year, representing the most frequent events during follow-up. Notably, only 32% of patients remained free from major adverse events at 2 years, decreasing further to just 15% at 5 years. These findings underscore that, despite substantial technological advances, long-term LVAD support continues to impose a considerable burden on both patients and healthcare systems.

Among device-related infections, 89% were driveline infections, which continue to represent a major Achilles’ heel of LVAD therapy. These observations reinforce the need for fully implantable systems or transcutaneous energy transmission technologies capable of eliminating the percutaneous driveline. Until such innovations become clinically available, meticulous outpatient follow-up, infection prevention strategies, and patient education remain essential components of successful LVAD care.

The burden of HF hospitalization is equally noteworthy and reflects the complex interplay between device-related factors, such as progressive aortic insufficiency, and patient-related factors, including progression of right ventricular failure, multiorgan dysfunction, arrhythmias, renal impairments, and recurrent volume overload. The study therefore reinforces an increasingly recognized paradigm: successful LVAD programmes require not only appropriate patient selection and surgical excellence but also well-coordinated longitudinal multidisciplinary care. This is consistent with recent population-based data demonstrating substantial healthcare utilization among LVAD recipients, including frequent hospital admissions and ongoing multidisciplinary care needs, despite improvements in device technology.^10^ Moreover, further gains in patient outcomes may increasingly come from advances in patient management, including improved prediction of right ventricular failure and refinement of anticoagulation strategies.^11–13^

Although the authors demonstrated significant and sustained improvement in New York Heart Association (NYHA) functional class despite the occurrence of adverse events, additional data regarding the long-term impact of these complications on functional capacity and quality of life would have been valuable in better defining the net benefit associated with LVAD therapy.^14^

One of the most sobering observations in this study is the prolonged transplant waiting time, with median waitlist durations exceeding 30 months. Such waiting periods are substantially longer than those currently reported, not only in many US programmes, particularly following the 2018 allocation policy changes, but also in several other European countries.^15^ Waiting times are highly relevant, as they profoundly influence the contemporary role and utilization of LVAD therapy across healthcare systems. In contrast to the USA, where LVAD implantation as BTT is often conceived as a relatively short-term strategy, the markedly longer waiting times and persistent donor shortage observed in many European countries increasingly transform LVAD support into a prolonged therapeutic phase even among actively listed patients.

In this context, the data reported by Legtenberg *et al*. are particularly informative. Despite the exceptionally long waiting times observed in this cohort, the majority of patients implanted as BTT or BTC ultimately remained suitable candidates for HTx. These findings highlight the important role of contemporary LVAD support not only in stabilizing patients with AdvHF but also in preserving end-organ function and maintaining HTx eligibility during prolonged periods on the waiting list.

Remarkably, despite 18.8% of patients having a history of HF shorter than 1 month and 31.2% shorter than 1 year, no patient underwent LVAD explantation due to myocardial recovery. This finding is somewhat surprising in light of previous data reporting myocardial recovery resulting in successful LVAD explantation in 3% and 1.4% of patients in the INTERMACS and EUROMACS cohorts, respectively, with rates rising to 40% in a highly selected non-ischaemic cohort undergoing a standardized mechanical and pharmacological unloading protocol. The absence of recovery-related explantation in such a highly specialized centre may reflect both the lack of a systematic recovery-focused strategy and the persistent uncertainty surrounding patient selection and long-term durability of myocardial recovery after LVAD support.

Several limitations warrant consideration. As acknowledged by the authors, this is a retrospective single-centre analysis and therefore susceptible to selection bias and residual confounding. Furthermore, outcomes in a medium-volume Dutch centre may not fully generalize to lower-volume institutions or healthcare systems with different transplant access and outpatient care infrastructures. However, these limitations do not diminish the study's relevance. On the contrary, real-world observational analyses are increasingly essential as LVAD therapy expands beyond tightly controlled trial populations.

## Declarations

### Data Availability

No data were generated or analysed for this manuscript.

## References

[1] Subramaniam AV, Weston SA, Killian JM, Schulte PJ, Roger VL, Redfield MM, et al Development of advanced heart failure: a population-based study. Circ Heart Fail 2022;15:e009218. 10.1161/circheartfailure.121.009218 35332793 PMC9117446

[2] Crespo-Leiro MG, Metra M, Lund LH, Milicic D, Costanzo MR, Filippatos G, et al Advanced heart failure: a position statement of the Heart Failure Association of the European Society of Cardiology. Eur J Heart Fail 2018;20:1505–35. 10.1002/ejhf.1236 29806100

[3] Guidetti F, Lund LH, Benson L, Hage C, Lindberg F, Basile C, et al Real-world prognostic performance of different severe and advanced heart failure definitions: data from the Swedish Heart Failure Registry. J Heart Lung Transplant 2026. 10.1016/j.healun.2026.03.008 41862040

[4] Baudry G, Crespo-Leiro MG, Delmas C, Guidetti F, Bravo MJB, Valente F, et al Identifying and overcoming barriers to referral in advanced heart failure. A scientific statement of the Heart Failure Association (HFA) of the ESC. Eur J Heart Fail 2025;27:3312–25. 10.1002/ejhf.70003 40819957 PMC12803578

[5] Veen KM, Ahmed M, Stark C, Botta L, Anastasiadis K, Bernhardt A, et al The fourth report of the European Registry for Patients with Mechanical Circulatory Support (EUROMACS) of the European Association for Cardiothoracic Surgery: focus on standardized outcome ratios. Eur J Cardiothorac Surg 2025;67:ezaf016. 10.1093/ejcts/ezaf016 39874447 PMC11879288

[6] Mehra MR, Uriel N, Naka Y, Cleveland JC, Yuzefpolskaya M, Salerno CT, et al A fully magnetically levitated left ventricular assist device—final report. N Engl J Med 2019;380:1618–27. 10.1056/NEJMoa1900486 30883052

[7] Legtenberg S, Ter Maaten JM, Erasmus ME, Kuijpers M, Douglas YL, Puspitarani RADA, et al Long-term outcomes of patients implanted with a HeartMate 3 left ventricular assist device-a real-world, single-centre, observational study. ESC Heart Fail 2026;13:xvag074. 10.1093/eschf/xvag074 41812231 PMC13036835

[8] Mehra MR, Goldstein DJ, Cleveland JC, Cowger JA, Hall S, Salerno CT, et al Five-year outcomes in patients with fully magnetically levitated vs axial-flow left ventricular assist devices in the MOMENTUM 3 randomized trial. JAMA 2022;328:1233–42. 10.1001/jama.2022.16197 36074476 PMC9459909

[9] Schmitto JD, Shaw S, Garbade J, Gustafsson F, Morshuis M, Zimpfer D, et al Fully magnetically centrifugal left ventricular assist device and long-term outcomes: the ELEVATE registry. Eur Heart J 2024;45:613–25. 10.1093/eurheartj/ehad658 38036414 PMC10959573

[10] Iwaszkiewicz P, Rakoczy K, Makiola M, Krupka D, Makowska P, Herbetko K, et al Platform for the monitoring and treatment of patients with long-term mechanical circulatory support: a pilot study. Kardiol Pol 2025;83:1413–5. 10.33963/v.phj.108672 40990557

[11] Livingston CE, Kim D, Serletti L, Jin A, Rao S, Genuardi MV, et al Predicting right ventricular failure after left ventricular assist device implant: a novel approach. ESC Heart Fail 2025;12:1916–31. 10.1002/ehf2.15200 39829406 PMC12055376

[12] Osman AF, Scott SS, Ezeh EO, Osuji E, Ailemen IO, Derbala MH, et al Role for inflammatory markers in predicting right ventricular failure in mechanical assist device recipients. ESC Heart Fail 2025;12:2608–20. 10.1002/ehf2.15160 40299892 PMC12287856

[13] Jdaidani J, Shadi M, Asogwa N, Winik J, Rossi D, Saikus C, et al Long-term outcomes of apixaban as main anticoagulant in patients with HeartMate 3 left ventricular assist devices. ESC Heart Fail 2025;12:1714–8. 10.1002/ehf2.15182 39654384 PMC12055345

[14] Slade AL, McMullan C, Haque MS, Griffith S, Marley L, Quinn D, et al Development of a quality of life measure for left ventricular assist device recipients using a mixed methods approach. ESC Heart Fail 2024;11:3167–79. 10.1002/ehf2.14850 38873750 PMC11424331

[15] Maitra NS, Dugger SJ, Balachandran IC, Civitello AB, Khazanie P, Rogers JG. Impact of the 2018 UNOS heart transplant policy changes on patient outcomes. JACC Heart Fail 2023;11:491–503. 10.1016/j.jchf.2023.01.009 36892486 PMC12812422

